# Implementation Challenges and Recommendations for Employing Peer Support Workers in Emergency Departments to Support Patients Presenting after an Opioid-Related Overdose

**DOI:** 10.3390/ijerph19095276

**Published:** 2022-04-26

**Authors:** Annette S. Crisanti, Jennifer Earheart, Megan Deissinger, Kathryn Lowerre, Julie G. Salvador

**Affiliations:** 1 Department of Psychiatry and Behavioral Sciences, University of New Mexico, Albuquerque, NM 87131, USA; jennifer.a.earheart@gmail.com (J.E.); jgsalvador@salud.unm.edu (J.G.S.); 2 Epidemiology and Response Division, New Mexico Department of Health, Santa Fe, NM 87505, USA; mdeissing@phs.org (M.D.); kathryn.lowerre@state.nm.us (K.L.)

**Keywords:** peer support workers, opioid use disorder, emergency departments

## Abstract

The placement of a peer support workers (PSWs) in emergency departments (ED) is a promising practice for supporting persons with opioid use disorder who are presenting with an overdose or related medical condition. However, this practice is underutilized. The objective of this study was to identify the challenges of employing PSWs in the ED and provide a checklist to increase the likelihood of their successful integration and retention in this environment. Qualitative methods were used to collect data from nineteen key stakeholders who worked in hospital settings. Using a social-ecological model, themes were identified at the system, hospital, and individual levels. To support integration of PSWs and buy in for the ED team, our findings indicate a need for a planning phase that includes collaboration between leadership, ED staff, and PSWs. Specifically, planning should address four areas: (1) hiring a PSW that is a good fit for the fast-paced ED setting, (2) education of ED staff on the value and role of PSWs, (3) establishing workflow protocols, and (4) providing PSWs with training and appropriate supervision.

## 1. Introduction

An estimated two million people have an opioid use disorder (OUD) [1]. In 2019, almost 50,000 people died from overdoses involving opioids, including prescription pain medications, heroin, and synthetic opioids [2]. Recently, many western states have reported massive increases in overdose deaths. From 2018 to 2019, the largest increase in death rates involving synthetic opioids, such as fentanyl, occurred in the western U.S. (67.9%) [3]. Between May 2019 and May 2020, over 81,000 drug overdose deaths occurred in the U.S., the highest number of overdose deaths ever recorded in a 12-month period, suggesting an acceleration of overdose deaths during the pandemic [4]. During this time period, 10 western states reported over a 98% increase in overdose deaths: Alaska, Washington, Oregon, California, Nevada, Arizona, New Mexico, Colorado, Oklahoma, and Texas [5].

Between 2016 and 2017, the United States reported a 29.7% increase in the number of emergency department (ED) visits for opioid overdose [6]. Risk of relapse is particularly high among those with OUD who are being discharged from prisons, inpatient units, and detox centers [7]. In New Mexico (NM), there was an 106% increase in the rate of opioid overdose emergency department visits from 2014–2018 [8]. In 2018, the rate of ED visits, related to opioids, in NM was 61.1 per 100,000, reaching as high as 183.7 per 100,000 in one county [8].

With the rise of opioid overdose ED visits, there is a need to make this setting “a critical entry point for primary and secondary prevention of opioid overdose” [9] (p. 689). When patients present to EDs with an opioid overdose or opioid-related event (e.g., abscess) the goal is to get individuals stabilized and discharged as efficiently as possible. In some cases, a patient may be discharged with information on addiction resources, but referral to medication-assisted treatment and follow-up are less common, especially in rural or remote areas [10,11]. Consistent guidelines for post-care following an overdose are also limited. Vivolo-Kantor et al. recommend emergency departments establish “post-overdose protocols that can help prevent subsequent overdose by providing naloxone and connecting patients with case management services or peer navigators to help link them into treatment and harm reduction services” [6] (p. 284). Studies have also shown that patients are more likely to engage in treatment and reduce their self-reported opioid use when medication-assisted treatment is initiated in the ED [12].

Peer recovery support services have been shown to be effective in increasing social supports, patient engagement, and well-being [13,14,15]. Peer support workers (PSWs), also known as peer specialists, recovery coaches, or peer advocates, are individuals with lived experience with mental illness and/or substance use disorders who are in recovery and deliver services in healthcare settings. The provision of peer recovery support services is an established component of recovery-oriented care. Recently, peer recovery support services have been implemented in some EDs in the United States, specifically in response to the opioid epidemic [16,17,18,19]. Because of their lived experience, particularly with substance use, PSWs are able to better connect with people at the time of crisis. The limited body of evidence indicates that PSWs in EDs have resulted in increased linkage to care, shorter days to initiation for substance use treatment, improved engagement with high-risk populations, increased harm reduction education, and provision of naloxone [20,21,22,23]. However, there is still limited published research regarding the feasibility, acceptability, and effectiveness of the role of PSWs supporting patients with OUD in the emergency department setting [24].

One critical factor, regarding the implementation of peer recovery support services in EDs, is how to best incorporate a PSW. Preparing PSWs and ED staff for successful implementation and sustainability is critical, given the nature of the setting, with high stress, long shifts, and frequent exposure to potentially traumatic events [25,26]. High workloads and emotional drain are key stressors for staff in EDs, leading to a moderate-to-high level of burnout among physicians and nurses [27,28,29]. It is, therefore, useful to understand ED challenges and provide guidance for hospitals to consider, prior to implementing peer recovery support services.

The objective of this study was to identify the challenges to implementing peer recovery support services in the ED setting, to support patients who present with opioid overdose or another opioid-related emergency need. Specifically, these challenges were identified from the perspective of key stakeholders working within hospitals that were currently employing peer recovery support services and considering this model, but not yet implementing it. In addition, perspectives were obtained from a group of experts in the employment of PSWs in hospital and other behavioral health settings.

## 2. Materials and Methods

**Research Purpose.** In 2018, the NM Department of Health received funding through the Centers for Disease Control and Prevention’s (CDC) Cooperative Agreement for Emergency Response to address the opioid overdose epidemic, aimed at advancing our understanding of the opioid overdose epidemic and scale-up prevention activities across all 50 States and Washington, D.C. [30]. With these funds, the NM Department of Health (DOH) sought to enhance and expand an intervention aimed at addressing non-fatal opioid overdose admissions by incorporating one-on-one peer recovery support services into EDs in four NM counties. To support the successful implementation of this intervention, the DOH contacted the lead author (AC) to design and conduct a qualitative study, to identify the challenges and related guidance for implementing PSWs in the ED. AC had previous (1) expertise in research involving PSWs in behavioral health settings at the state, as well as doctorate level; (2) training in qualitative research; and (3) expertise implementing qualitative research. AC then engaged a second colleague (JE), as well, with master’s level training in qualitative research. Rather than testing a known theory, we used an inductive approach examining the qualitative data to reveal the important issues identified by hospital leadership, staff, and PSWs.

The primary goal of this study was to identify challenges with implementing peer recovery support services in ED settings to support persons with OUD. Using an inductive approach, these challenges were organized under overarching categories and themes. After analysis was completed, the researchers used a modified social-ecological framework to help convey the relationship between main theme categories, emphasizing the interrelated nature of the challenges identified at the individual, hospital, and system levels [31]. Tips to facilitate the integration and retention of PSWs within EDs are presented in a checklist and discussed.

**Participant Selection.** A total of 19 key stakeholders were contacted to participate in qualitative data collection because of their hospital-related roles and expertise in the delivery of peer recovery support services or with directly supervising or employing PSWs in ED settings. The primary author identified six content experts with a long history of supervising and employing PSWs, who were known to the lead author (AC) from her experience in mental health services research in NM. With the assistance of the NM DOH, eight persons in clinical director or management positions were identified at the hospitals that were contracting with them to incorporate peer recovery support services into their EDs. Last, snowball sampling was used, which asked both the content experts and clinical directors to identify other persons in these hospitals who had expertise with PSWs and/or working in EDs [32]. Through this process, an additional two nurses and three PSWs were identified. Snowball sampling ceased when the authors determined that there was enough data to develop a robust and valid understanding of the study phenomenon, specifically operationalizing this as data saturation, reflecting the degree to which new data repeated what was expressed in previous data [33].

**Data Collection.** Respondents were given the choice to participate in either a semi-structured 1–1 interview, paired depth interview (involving one interviewer with two persons from the same agency), or an in-person focus group. Participants who selected an interview format (1–1 or paired) could choose to respond via telephone or in person. A draft of the interview/focus group guide was developed by the research team, led by co-authors (AC and JE). To strengthen the validity, the draft was shared with experts, including local PSWs, for cognitive testing of the questions, to help ensure the questions were measuring the intended phenomenon (challenges of implementing PSWs in the ED) [34,35]. Open-ended questions included, for example, “What current protocol is in place for patients presenting with an overdose?” and “Can you tell me more about the process or protocol for incorporating PSWs in the provision of services in the ED?” The second author (JE) contacted identified key stakeholders to invite them to participate. Data collection was audio recorded whenever possible (in the majority of instances), and detailed notes were taken using active listening skills to repeat back responses, in order to help ensure accuracy and as a back-up for any poor audio recordings. Verbal consent was obtained, and participants were not compensated for their time. Transcribed interviews or detailed notes (for non-recorded instances) were the source for the content analysis, as described below. This study was approved by the lead author’s university’s human research protections office (ID# 20-244).

**Data Analysis Method.** The analytical approach was inductive and used content analysis to code data and identify overarching themes directly from the text [36,37]. Our decision to use content analysis was aimed at allowing the respondents’ own words from the transcribed texts to be the direct basis for coding and theme development. Furthermore, our approach was best aligned with what is called “manifest” content analysis, where the researcher “stays very close to the text, uses the words themselves, and describes the visible and obvious in the text.” [38] (p. 10). To enhance the reliability of our findings during the analysis, the development of codes, themes, and labels followed an iterative process between the primary coders (AC and JE) to ensure agreement, followed by discussion of codes and themes with the entire authorship team before finalization. Using printed copies of each data collection event (n = 13), the first two authors (AC and JE) independently read participants’ responses and independently developed the initial code names (i.e., labels), to help reduce the data into meaningful coding categories. This was done using pen/paper and written directly onto the transcripts. After, the authors met in person to discuss their coding categories, coming to agreement on each and documenting this in the transcribed texts. Then, the authors developed a codebook, separately returned to the documents, and independently coded each again, following the developed codebook. Intercoder agreement was assessed by comparing the coding for each document and discussing any differences, until joint agreement was obtained. In cases where the authors coded differently, they referred back to the text to discuss the exact statement and come to agreement on the simplest manifest meaning, which was intended to resolve differences. There were no major disagreements or conflicts in the coding. Last, the first two authors organized the codes into themes and appropriate labels through discussion and reflection on the coded data. These codes and themes, including example text, were shared with co-authors (MD and KL), who had insight into the subject matter and were familiar with the key stakeholders and agencies that they represented, for their input and agreement. All manuscript authors agreed with the overarching themes and final labels chosen.

## 3. Results

### 3.1. Characteristics of Study Subjects

All 19 persons contacted about the study agreed to participate. Seven respondents came from hospitals that had incorporated peer recovery support services in the ED, seven respondents were from hospitals that were planning to do so (but had not yet started), and five respondents came from non-hospital settings, but had ample expertise in providing and employing PSWs. The hospitals were mainly located in urban areas. Data collection lasted between 60 to 75 min and was audio recorded and transcribed, except for four interviews. These four interviews were conducted without a recorder because the setting was not conducive to quality audio recording (public or outdoor space), while communication between interviewer and respondent remained clear. Given the variety of respondents and their choice of data collection type and mode, Table 1 provides the full details for each respondent, setting, and data collection.

### 3.2. Identified Themes

**Figure 1:**
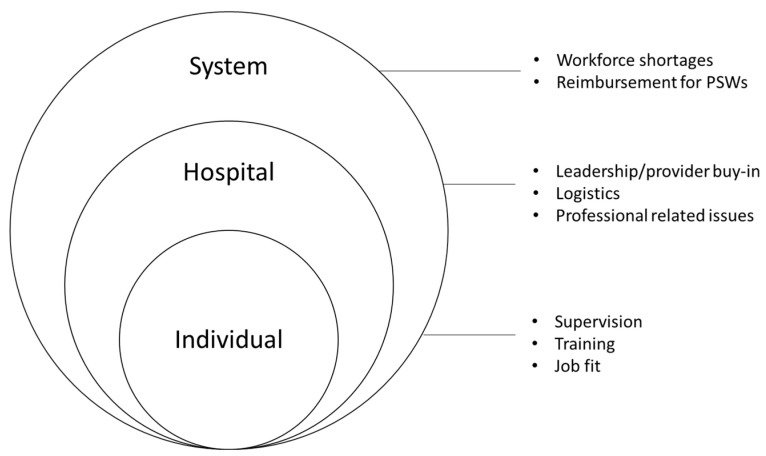
Implementation challenges identified by key stakeholders in employing peer support workers (PSWs) in the emergency department to help care for patients that present after an opioid-related overdose.

Key stakeholders identified several challenges to incorporating PSWs within the ED. These themes and selected representative samples of qualitative responses are presented in Table 2 and were agreed upon by all authors. There were three main theme categories identified: system-, hospital-, and individual-level challenges. Themes at the system level were PSW workforce shortages and reimbursement challenges. Themes at the hospital level were the need for buy-in of hospital providers and staff, logistics related to integrating PSWs, and concerns for the professionalism of PSWs. Themes at the individual level were the need for appropriate supervision, PSW training, and selecting a PSW that is a good fit for the ED environment. Figure 1 presents a modified social-ecological model [31] to emphasize the relationship between system-, hospital-, and individual-level challenges. The overlapping rings emphasize that, for the employment of PSW in the ED to be most successful, it is important to address challenges across all levels simultaneously. The overlapping rings in the model also highlight that challenges may pertain to more than one level. For example, supervision and training (which we have grouped under individual-level challenges) could also be considered hospital-level challenges, in that it would be the hospitals’ responsibility to provide supervision and training to support PSWs. Our decision to group supervision and training under individual-level challenges was based on the context in which these challenges surfaced, as noted by the respondents in the interviews.

## 4. Discussion

Several of the concerns revealed in our research reflect those that have been previously identified in the literature, including the importance of training of ED personnel about PSWs, clarifying the PSW role in the ED, obtaining buy-in, ensuring the PSW is a good fit, collaboration with a recovery community organization, privacy concerns, and payment/reimbursement [16,17,18,19,24,26,39]. Based on the study results, the authors of this manuscript have provided a checklist (see Table 3) describing the key areas for consideration, to help hospital EDs successfully hire and integrate PSWs. These are suggestions based on the authors’ reflections on the main themes presented in Table 2 (e.g., workforce, reimbursement, buy-in, etc.), in addition to their experience employing PSWs and implementing peer recovery support services in various environments (including the ED).

Highlights of Table 3 are presented below.

### 4.1. System Level

Workforce: A key area is recruiting and hiring a PSW who is prepared for ED work. Successful strategies include providing competitive compensation and benefits, to attract a large enough pool of applicants, from which to interview and select a PSW that will be a good fit. A survey of PSWs in Georgia found that PSWs were likely to be unemployed, and those that were employed were in positions with limited benefits and low income, i.e., $10,000–20,000 [40]. Other strategies to address workforce challenges include working with a recovery community organization that can directly employ PSWs and has knowledge of a large pool of PSWs, knowledge of where to advertise and recruit PSWs, and can provide other important functions (e.g., supervision and training, discussed further below).

Reimbursement: As with any hospital staff, understanding how to reimburse for peer recovery support services is critical for the success. One strategy is to explore reimbursement possibilities (e.g., U.S. Medicaid or other federal government insurance programs for low-income adults and people with disabilities outside of the United States). In the U.S., this includes Medicaid 1115 waivers and developing Medicaid state plan amendments [16]. At the same time, hospitals can consider the potential cost savings for hospitals who implement peer recovery support services. For example, PSWs can help reduce “high utilizers” of EDs, particularly patients with OUD and co-occurring disorders that rely heavily on emergency services for their healthcare. A study, conducted in Delaware, found that patients who engaged in a brief intervention (which included motivational interviewing) led by a PSW had improved healthcare utilization, which could be correlated to cost savings. In one cohort, patients who were connected to substance use treatment through a PSW had a 58% decrease in inpatient medical admissions ($68,422), 13% decrease in ED visits ($3308), 32% decrease in behavioral health inpatient admissions ($18,119), and 32% decrease in outpatient admissions ($963). Among this cohort of 25, this represents a $88,886 difference in healthcare costs [41].

### 4.2. Hospital Level

Buy-in: For ED directors/managers and staff to buy into the idea of employing a PSW, it is important that they have a full understanding of what PSWs do and how best to integrate them within the ED [39,42]. Research demonstrates that conflicts can arise when staff are not prepared for the inclusion of PSWs [30]. Increasing support from staff and providers can increase the likelihood of PSW success. Richardson and Rosenberg [16], as well as Gates and Akabas [43], suggest that strategies to build relationships between PSWs and other staff focus on effective communication related to patient cases and opportunities to increase mutual understanding and support. 

Logistics: A useful approach to support the logistical details of PSW integration (where peer will be stationed, who will they report to, how will they be contacted to connect with a patient, etc.) is through a memorandum of understanding with a recovery community organization [16]. These organizations have knowledge of PSWs and their role in recovery, and they can help provide appropriate supervision and education for hospital providers and staff on the role of PSWs. Further, they can employ PSWs directly, which often provides more flexibility for the hiring of persons who may have criminal backgrounds related to their substance use disorder. Hospitals that choose to hire PSWs directly, rather than via a recovery community organization, may find this is facilitated by the hospital’s prior experience with PSWs, having the capacity to train staff about PSWs (and train PSWs in the ED processes), and access to appropriate PSW supervision, as well as a plan for reimbursement. Direct hiring of PSWs is sometimes the only option if there is no recovery community organization locally; however, virtual consultation with a recovery community organization to support integration may be possible and fruitful. For identification of other logistical issues related to the implementation of mobile recovery outreach teams for opioid overdose patients in the emergency room, see reference [17].

Concerns Related to Professionalism/Relapse: Hospital staff and provider concerns, related to professionalism, may be addressed, in part, through awareness of training obtained by PSWs, as a part of their preparation for employment that is required by recovery community organizations and/or regulatory bodies. This training is expected to focus on the Health Insurance Portability and Accountability Act and professionalism broadly (dependability, dress, demeanor, diplomacy, and discretion). PSWs that have been certified as PSWs can provide documentation of the training they have received and areas the training covers, with more detail available from the agency providing the certification. With specific respect to concerns about relapse, Chinman et al. argued that there is “no evidence that the demands of work exacerbate health conditions or lead to relapses among peer specialists” [44] (p. 21). In fact, meaningful, competitive work may serve to enhance recovery. Furthermore, research indicates that employment is linked to beneficial effects on a peer’s clinical and social functioning [45]. Furthermore, according to a technical guide for clinical staff on how best to integrate consumer providers into staff culture, “the persistent misconception that consumer providers will inevitably relapse should be addressed and dispelled” [44] (p. 17). However, a relapse can happen. Therefore, as with all employees, PSWs should have access to wellness resources within an agency and be encouraged to confide in their supervisors when their symptoms are becoming symptomatic; supervisors should be encouraged to tactfully point out behaviors that PSWs may be exhibiting that may be of concern.

### 4.3. Individual Level

Appropriate supervision is key to the success of a PSWs. The document entitled Pillars of Peer Support Supervision is a useful resource for guiding integration of peer recovery support services, and it is publicly available [46]. This document details the following key points: ensure supervisors (1) are trained in quality supervisory skills, (2) understand and support the role of the peer specialist, (3) understand and promote recovery in their supervisory roles, (4) advocate for the peer specialist and peer specialist services across the organization and in the community, and (5) promote the professional growth of the peer. Again, recovery community organizations can support selecting an appropriate supervisor and may be able to provide this service directly.

Need for Additional Training: Providing additional training in provider and staff roles, policies and procedures, and similar basic onboarding is important for PSWs, as well as providing other training opportunities for professional development that can improve their employment experience [40]. Remembering to include PSWs in general staff training events can be an easy approach to help PSWs and other staff feel part of the same ‘team’ and increase the PSWs’ skill set. For example, Crisanti et al. found that PSWs and clinical providers benefited equally from a one-day training in the evidence-based, behavioral health-focused Seeking Safety© program and found similar outcomes, in terms of satisfaction and comfort level in implementing this evidence-based practice [47]. As with all ED staff and providers, it is important that PSWs have role clarity, as part of their training and onboarding. “Poorly defined job descriptions make it difficult for PSWs to be successful and hinder their integration into multi-disciplinary work teams” [48] (p. 2). In interviews with PSWs in Canada, participants noted there was a disconnect between the training they received and stressful nature of their work [42]. Choosing a PSW that is a Good Fit for the ED Environment: It is helpful to match an employee’s strengths, needs, and experience to job requirements and work environment, to support overall satisfaction and mental and physical well-being [49,50]. Because of the nature of the work environment, those working in an ED must have superior coping skills and a high level of self-management, which involves taking an active role in one’s recovery and wellness [26]. PSWs hired to work in EDs should be well-established in their recovery and have a strong sense of resiliency. Self-care is especially important

Limitations: Only one respondent was from a rural location, limiting the application of findings for rural hospitals. While the relatively small sample size in this study is common with qualitative research, the generalizability of the findings is limited. However, the qualitative approach was used purposefully to detail particular viewpoints and experiences, regarding the implementation of PSWs. Another limitation is that physicians were not identified in the recruitment processes, including chain-referral. Inclusion of their views would strengthen study findings. However, the inclusion of other ED clinical staff, including directors, managers, and nurses, as well as PSW experts, is an important contribution to the literature, as these staff work closely with PSWs in the ED setting. The input that we received from PSWs themselves was especially informative. Audio recording was not used for four interviews, due to settings not conducive to recording. However, detailed notes were taken in those instances. It is noteworthy that research has shown comparable data quality between audio-recorded transcripts and high-quality interview notes [51]. While a strength overall in guiding this study, the previous experience of the lead author (AC) conducting research with PSWs presents a potential source of bias. AC has published on successful implementation of peer support models and guidance on hiring and employment of PSWs. During the design and analysis phases, the selection of manifest content analysis and independent coding of the texts were steps to address bias and help ensure respondents own words were the source for overall codes and themes. Furthermore, the review and discussion by all authors helped further minimize bias. The participants engaged in the interviews in an official capacity, as part of their paid work hours, which may have influenced their choice to participate and insight shared with the research team. Finally, the analysis was focused on identifying barriers in the whole sample, not comparing across contexts (e.g., barriers in different EDs or those identified in ED’s implementing PSWs and those in planning stages). Conducting a larger study, comparing responses of EDs at different phases (contemplation, adoption, implementation, maintenance, etc.), would be useful for future research.

## 5. Conclusions and Implications for Clinical Practice

PSWs can play an important role in the care of patients that present to the ED after an opioid-related overdose or related emergency (e.g., abscess). In doing so, peer recovery support services can help address the opioid crisis by introducing patients to harm reduction medications (naloxone), treatment options (medications for opioid use disorder), and link persons to community-based treatment and recovery resources. Planning should include thoughtful conversations between leadership, ED staff, and PSWs, as well as a commitment from leadership and hospital staff to recovery-oriented care. Planning should focus on, but not be limited to, four key areas: (1) hiring the right PSW for the position, (2) education of ED and hospital staff on the value and role of PSWs, (3) establishing workflow protocols, and (4) providing PSWs with training and supervision. Recognizing challenges to PSWs employment and implementing strategies to address these challenges throughout planning and implementation can increase the likelihood of successful integration and retention of PSW into EDs. With respect to implications for clinical practice, the education of the entire ED staff, regarding the position and role of PSWs, is critical [39]. Without full understanding of the position and role, conflicts are likely to arise and potential failure for peer recovery support services in that setting. This can further create a barrier where PSWs, ED providers, and other clinical staff reject peer recovery support services inherently, when, in reality, the model was improperly implemented. Similar clinical failures can result from not understanding other factors raised in the present study, including the need for appropriate PSW supervision and issues with employment and sustainability. The checklist is intended to be an implementation guide to be applied in clinical practice, to increase the likelihood of successful integration and retention of PSWs in the ED.

## Figures and Tables

**Table 1:** Key stakeholder role, setting, and data collection details.

Respondent Role	Hospital Had PSW?	Data Collection	Format	Mode
Manager	Yes	1–1 interview	Phone	Recorded
2. Director	Yes	Paired interview	Phone	Recorded
3. Manager	Yes			
4. Director	No	Focus group	In-person	Recorded
5. Director	No			
6. Manager	No			
7. Manager	No			
8. PSW	No			
9. PSW	Yes	1–1 Interview	Phone	Recorded
10. PSW	Yes	1–1 Interview	Phone	Recorded
11. Nurse	No (rural) *	1–1 Interview	Phone	Recorded
12. Nurse	No	1–1 Interview	Phone	Recorded
13. Manager	Yes	1–1 Interview	Phone	Recorded
14. Content Expert	N/A ^+^	1–1 Interview	Phone	Recorded
15. Content Expert	N/A ^+^	1–1 Interview	Phone	Notes
16. Content Expert	N/A ^+^	1–1 Interview	Phone	Notes
17. Content Expert	N/A ^+^	Paired interview	Phone	Recorded
18. Content Expert	N/A ^+^			Recorded
19. Content Expert	Yes	1–1 Interview	In-person	Recorded
**Summary of Data Collection Details**				
Manager/directors (8)	Hospitals w/PSW (7)	1 Focus group (N = 5)	Phone (N = 13)	Recorded (N = 15)
Nurses (2)	Hospitals no PSW (7)	2 paired interviews (N = 4)	In-person (N = 6)	Notes (N = 4)
PSWs (3)	Participant not from	10 1–1 interviews (N = 10)		
Content experts (6)	hospital setting (5)			

* One participant from rural setting; all others urban setting; PSW = peer support worker. + Participant not from hospital setting.

**Table 2:** Summary of results: challenges to implementing Peer Support Workers (PSWs) in emergency department settings.

System-Level Themes	Respondent Statements
PSW workforce shortages	*The primary issue we had with hiring for the position was that we had a ton of folks who applied for the position without meeting the position qualifications. … We had a lot of people who were informal peer support workers but didn’t have the certification.* *I personally don’t feel like these folks are making enough money. I really don’t think it’s an appropriate wage for the type of work they are doing.*
Reimbursement for peer services	*We run a tight ship and there’s not a lot of money to bring in other staff and so that’s a real valid concern and I’d say that’s true for all rural hospitals. We are staffed to a minimum core.* *The major challenge is going to just be finances in general. We’re constantly having to keep an eye on our productivity. So adding another staff position without taking away a position that we already have filled would be the huge challenge.*
**Hospital-Level Themes**	
Buy-in from hospital leadership, providers, and staff	*Selling our staff on the value and utilizing peer in the right ways would be the initial challenge.* *I think there’s not a lot of understanding about what a PSW is. I don’t know the exact role. I think there would be a lot of conversation about function between our counselor in the ED, social worker inpatient, and then how we would work as a whole team and what that would look like….* *Be specific about the role of the PSW including job expectations, requirements, and specific duties.*
Logistics related to integrating PSWs	*It’s so ebb and flow. We don’t know. We can’t predict who’s going to walk in the door. And so it doesn’t make sense to have a PSW here all of the time…* *It’s important to have a clear process established on both sides so that everyone knows what is supposed to happen when a PSW is contacted. Otherwise, how are the ED staff going to know or remember in the chaos of everything going on…?*
Concerns related to professionalism	*[There are] worries that the peer would come in and in a small community like ours, whether there is going to be ‘chitter-chatter’ out in the community.*
**Individual-Level Themes**	
Need for appropriate supervision	*It’s so important to have good supervision. Having a plan for what to do if you feel overwhelmed is important and the peer support worker needs to feel comfortable with this person.*
Need for additional training	*Current CPSW training does not include enough information on reporting or documentation [which] can be challenging for peers, especially in the ED.*
Choosing a peer that is a good fit	*Identifying the correct peer for the emergency room is the most difficult part of this endeavor.* *If a PSW has a background in substance use, going to the ED when someone has OD’d can be really triggering.*

**Table 3:** Checklist for successful integration of Peer Support Workers (PSWs) into emergency department (ED) settings.

**System Level**	
Workforce	Clearly defined job description, so that PSWs applying for the position know what is expected. Possible PSW criminal background. Discussions with human resources around why hiring a person with “lived experience” is important may be warranted. Partner with a recovery community organization for employment. Participate in job fairs and offer limited shadowing experiences to introduce PSWs to ED environment. Attractive salary and benefits packages can increase the applicant pool and chance of finding the right PSW for the job.
Reimbursement	Understand billing codes that will enable reimbursement for peer recovery support services and sustainability. Medicaid 1115 waivers and amendments to Medicaid state plans. Understanding how PSWs may help reduce high-utilizer costs.
**Hospital Level**	
Buy-in	Training for ED providers, staff, and admin regarding PSWs, to include: the role of PSWs (e.g., job expectations, requirements, and specific duties), how PSWs will integrate with ED team, value added (including how PSWs can help with challenging or frequent utilizers), and how PSWs will engage and link patients to community-basedtreatment.
Logistics	Consider partnering with a recovery community organization to help develop protocols and workflow related to PSWs. Create a clear plan for how PSWs will respond to patients with OUD. For example, will the PSWs be contacted by ED staff or stationed on-site? Decide if the PSWs will be tasked with following-up with patients (for how long and what format (phone, in person, etc.).
Professionalism concerns	Highlight components (e.g., professionalism and confidentiality) that are included in most PSW certification programs. Address myths related to PSW relapse. Ensure PSWs have access to wellness resources; open communication with supervisor.
**Individual Level**	
Supervision	Recovery community organization or supervisor with experience with PSWs, supervisor must have strong supervisory skills, and be able to advocate for PSWs and peer recovery support services.
Training	Additional training for PSWs in the hospital and ED environments (policies, protocols, workflow, staffing, roles, etc.), as well as other areas related to professional development. Important for PSWs to receive training specific to the stressful and often high-pressure ED environment.
Good fit	PSWs comfortable working with multi-disciplinary teams, able to multi-task and remain calm amidst chaos, superior coping skills, high-level of self-management, and well-established in their recovery Exposure to ED in job fair and/or training is helpful.

## Data Availability

Not applicable.
